# Conditional Control of Instrumental Avoidance by Context Following Extinction

**DOI:** 10.3389/fnbeh.2021.730113

**Published:** 2021-09-28

**Authors:** Vincent D. Campese, Lauren A. Brannigan, Joseph E. LeDoux

**Affiliations:** ^1^Department of Psychology, University of Evansville, Evansville, IN, United States; ^2^Center for Neural Science, New York University, New York, NY, United States; ^3^Emotional Brain Institute, Nathan Kline Institute for Psychiatric Research, Orangeburg, NY, United States

**Keywords:** extinction, avoidance, renewal, context, instrumental

## Abstract

Using rodents, three training arrangements (i.e., ABB vs. ABA, AAA vs. AAB and ABB vs. ABC) explored whether extinction influences the expression of avoidance in a manner controlled by context. Retention testing following extinction showed that more avoidance responding (i.e., renewal) was observed when extinguished cues were tested outside of the context where they had undergone extinction. In contrast, response rates were significantly lower when stimuli were tested within the context where extinction learning had occurred. These findings add to the emerging literature assessing the role of Pavlovian extinction processes in the development of instrumental avoidance responding by demonstrating conditional control over extinguished responding by context. This study was conducted using a within-subjects approach that minimized the potential for context-outcome associations to bias responding, and thus, reflects hierarchical control over behavior based on the specific associative status of each tested cue in each training context.

## Introduction

For decades, aversive Pavlovian conditioning has been an effective model for human fear and anxiety (Lissek et al., 2005). Using this procedure great advancements in the understanding of the neurobiology that regulate behavior in aversive motivation have been made (Rajbhandari et al., 2017). Additionally, potential clinical applications to treat patients that suffer from maladaptive fear and anxiety, such as extinction have been explored using this framework (Morgan and LeDoux, 1995; Quirk et al., 2000; Maren and Holmes, 2016). While research into extinction has produced important findings about how conditioned aversive behavior can be attenuated, it has also demonstrated that extinction is not as effective of a treatment as desired (Bouton et al., 2021). After extinction, conditioned responding recovers following a variety of manipulations, including presentations of the extinguished stimulus in a different context or at a different time (Bouton, 2004). Thus, many researchers have begun to revisit other means capable of reducing defensive responding, such as avoidance (LeDoux et al., 2017; Cain, 2019). While some clinical disorders are defined by the perseveration of maladaptive avoidance, in other cases, avoidance behaviors can be adaptive and pro-survival. For example, during avoidance learning in rodent studies, maladaptive defensive responses (e.g., conditioned freezing/fear) are reduced and gradually replaced with proactive instrumental avoidance responding (e.g., shuttle or lever-press responding) to keep the subject safe and prevent harm. However, the interdependent nature of instrumental avoidance behavior and aversive Pavlovian extinction processes is difficult to disentangle. As such, an analysis of how the later contribute to former has not been sufficiently addressed to establish avoidance as any more effective than extinction in providing an enduring treatment for patients. Therefore, the current study explored the impact of extinction on the contextual control over signaled avoidance in rodents using a design in which renewal was measured in ABA, AAB and ABC conditions.

## Methods

### Subjects

Forty-eight male Sprague-Dawley rats were used as subjects in the study reported below. Rats were bred by and obtained from Hill Top Lab Animals (Scottsdale, PA, USA). Subjects weighing between 250 and 300 g at the start of experimentation were housed individually in ventilated, free-hanging plastic tubs and provided with free water and standard lab chow. The colony was maintained on a 12-h light/dark cycle and the study was conducted in compliance with and according to the guidelines of the Guide to the Care of the Use of Laboratory Animals of the National Institutes of Mental Health. Institutional animal care and usage committee (IACUC) approval for the procedures employed in this study was obtained through the New York University Animal Welfare Committee.

### Materials

All phases of the study were conducted using two-way shuttling chambers (model: H10-11R-SC) manufactured by Coulbourn Instruments (Allentown, PA). Over the course of the experiment, these chambers were manipulated to form distinct environments to study how context contributes to avoidance behavior. Each rectangular shuttle box was constructed of Plexiglass in the front and back and metal on the sides (50.8 x 25.4 x 30.5 cm; length x width x height) and were divided in half along the length of the chamber. The front and back walls were made of clear plexiglass and the side walls were made of a metal alloy. A metal divider with an opening (8 x 9 cm, width x height) cut in the center was positioned along the midline of the box, allowing the rat to move freely from side to side. The original shuttle box floor consisted of a series of electroconductive stainless-steel bars.

Each shuttle box was housed inside of a sound-attenuating chamber (Coulbourn Instruments, Whitehall, PA). Two speakers were mounted on opposite sides of the metal walls for delivery of the 5 kHz tone and 80dB white noise stimuli used in the study. A precision Animal Shocker (model H13-15-220; Coulbourn, Allentown, PA) delivered a 0.7 mA shock to the steel grid floors. Each chamber compartment was illuminated by two 5 W light bulbs on the top of the chambers. Shuttle responses (movement through the threshold between the two sides of the shuttle box) was registered via two infrared arrays. Each array was comprised of five emitter-detector pairs and located on either side of the midline divider. A desktop computer running GraphicState 3 (Actimetrics) software controlled the study, delivering stimuli and collecting behavioral data.

### Acquisition, Extinction and Test Contexts

**Figure 1:**
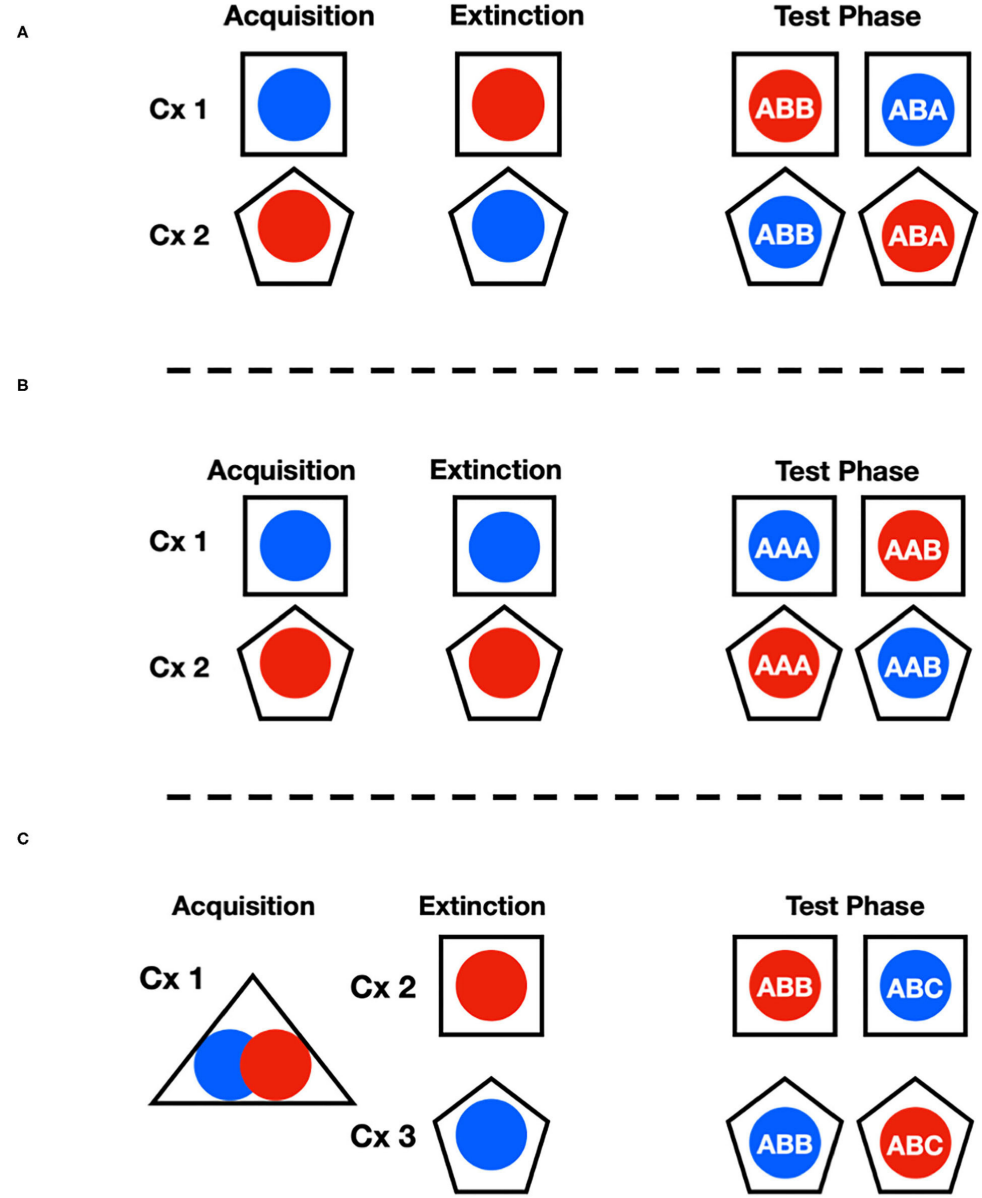
This figure shows the experimental design used in the three renewal conditions for ABA **(A)**, AAB **(B)** and ABC **(C)** renewal. The blue and red circles stand for CS1 and CS2 (tone and noise counterbalanced) while the square and pentagon shapes signify the different contexts. During the test phase, each cue presented in the different contexts produced the ABB vs. ABA as well as the AAA vs. AAB conditions. In **(C)**, it is shown that to produce the balanced contextual conditions to generate ABB vs. ABC renewal, both CS1 and CS2 were trained in the same context and then treated in separate contexts beyond that. It should be noted that since there were only two sets of chambers, this was accomplished by splitting context A across both rooms to ensure subjects were trained in physically different chambers in addition to the measure taken to facilitate perceptual distinction between the environments.

Two different rooms, each containing four shuttle boxes (eight shuttle boxes total) arranged in similar fashions, were used to train, extinguish, and measure signaled-active avoidance behavior during the study. Distinct contexts were made for this experiment by manipulating these chambers' tactile, visual, and olfactory attributes. This was done in a way that produced a total of three different context arrangements that were used to study different forms of context dependent extinction. When modified to produce a distinct context, printed patterned paper (e.g., checkers, circles) was placed outside the Plexiglass walls. Additionally, potent hand soap (Dr. Bronner's: ~5 mL, either peppermint or lavender) was added to the waste trays to further distinguish the chambers from one another using the olfactory modality. Solid plastic floor inserts were also used as needed to further distinguish the contexts. ABA, AAB, and ABC renewal were measured in three separate groups of rats using a mixture of the alterations described above over the acquisition, extinction, and test phases of the study (see Figure 1). To be specific, one context was simply the basic avoidance chamber with no modifications. A second context was made by inserting checkered paper against the outer walls of the chamber and adding peppermint odor to the waste tray. The ABB vs. ABA and AAA vs. AAB arrangements used these two contexts. The third context used to create the ABB vs. ABC arrangement involved covering the floors with hard smooth plastic, adding circle patterned paper and using the lavender odor instead. It should be noted that for this study, since there were only two sets of chambers, subjects were run in a way to ensure that neither of these contexts was in the same physical box. Other measures were taken to help differentiate pre-session cues in this case, such as having ambient room lights on or off. It should also be pointed out, that this third context was not used as an acquisition context since the plastic floors prevented subjects from making contact with the grid floors. Thus, context A in this study was exclusively the basic chamber, and contexts B and C were the modified versions described above. This ensured that each test context was the location of extinction for one, but not the other stimulus in each renewal condition.

### General Procedure

The study consisted of three phases: (1) SigAA Acquisition, (2) Extinction, and (3) Retention Testing. Each subject was given training with two stimuli (noise and tone) and the capacity for each cue to elicit avoidance responding was evaluated and compared in two different test locations following extinction. The arrangements used produced three renewal conditions referred to as ABB vs. ABA, AAB vs. AAA, and ABB vs. ABC. These letters denote the context locations where the different phases of the experiment took place for each group as a function of the stimulus history across the study and not simply the physical setting. For example, the ABB vs. ABA subjects experienced conditioning of avoidance for each stimulus in distinct contexts (e.g., CS1 in context 1 and CS2 in context 2: see Figure 1). In contrast, extinction occurred in the opposite context for each cue (e.g., CS1 in context 2 and CS2 in context 1). Subjects were then tested with each stimulus in both contexts, so that each cue served the ABA as well as the control ABB role (Ji and Maren, 2005; Campese and Delamater, 2013). This arrangement eliminated potential context-outcome associations that could have biased responding to any given cue-context combination, and therefore, any observed differences in avoidance rates reflect conditional control over responding by context.

### SigAA Acquisition

During SigAA training, an auditory stimulus (noise or tone) was presented after a 5-min baseline and paired with footshock. Following an inescapable first trial (only in the first training session for each cue), rats had the opportunity to learn that a shuttle response through the midline of the box in response to the auditory conditioned stimulus (CS) would result in termination of the cue and prevention of the scheduled footshock unconditioned stimulus (US). If rats failed to shuttle during the 15-s auditory stimulus (tone or noise), then the scheduled footshock was delivered, which lasted a maximum of 15-s. US presentations could be escaped by shuttling after shock onset, but in the absence of a response the shock lasted for 15-s. In total, each SigAA training session consisted of 30 CS trials, with an inter-trial-interval (ITI) that averaged 120-s; a single session lasted no more than one h and 20 min (see Choi et al., 2010).

Rats were trained to avoid shock over a period of five days and were subject to two acquisition training sessions each day. For the ABA and AAB groups, these sessions were in different contexts, while for the ABC group they were conducted in the same context. In all cases, at least 2 h of rest time in the rodent colony was interpolated between these sessions. The order of these sessions was alternated to avoid any potential time of day associations and effects of circadian control over responding (Iordanova et al., 2008; Zhou and Crystal, 2009).

### Extinction

Rats underwent extinction training over a period of five days and were subject to two extinction training sessions per day, one in each training context with at least 2 h of rest time between sessions. Similarly, extinction training employed the same approach as acquisition training by alternating the order of session types on each day. Extinction protocols were similar to acquisition protocols, but with two critical differences: first, when the 15 s stimulus (tone or noise) was presented, it was not followed with a scheduled footshock (US) and, secondly, shuttle behavior did not terminate the audio stimulus. In total 30 CS-no US trials, with an ITI that averaged 120 s were presented in a single extinction session that lasted one h and 20 min. During extinction, each group had each cue extinguished in a different location (see Figure 1).

### Retention Testing

Rats were tested in two separate sessions following extinction training. One was conducted 24-h after extinction concluded, and the other a week later, to encourage response recovery. Each cue was tested in each test session with a block of 15 trials before moving to the other cue. The stimulus testing order was counterbalanced across test context. For the subsequent test session, the subject was placed in the alternative context and presented with the stimuli a second time, in the opposite order, in a counterbalanced fashion across each renewal group. The testing protocol was similar to the extinction protocol in that there were 30 CS-no US trials, with an average ITI of 120 s and each test session lasting one h and 20 min.

## Results

**Figure 2:**
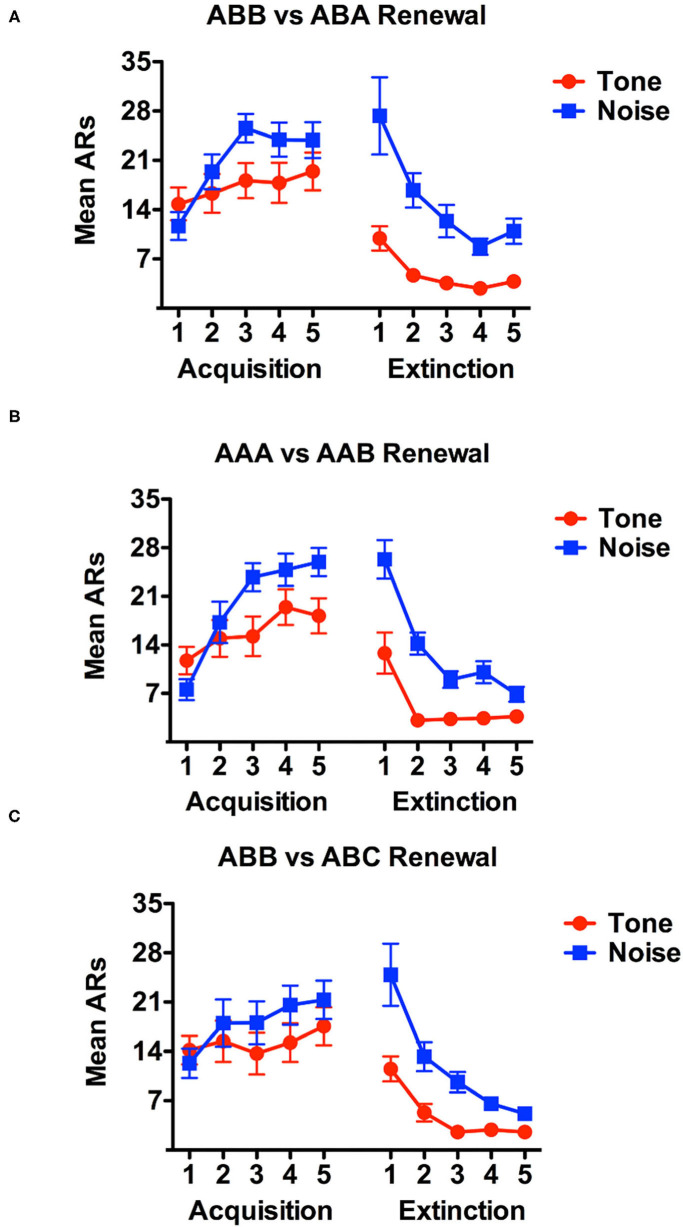
Acquisition and extinction data during the first two phases of the study are presented for each renewal condition in this figure. ABA subject data are presented in **(A)**, while AAB data in **(B)** and ABC in **(C)**. During acquisition, data reflect avoidance responses (ARs) only, meaning, shuttles that occurred during the ITI or during the shock on failed trials do not count toward this graph. During extinction, all shuttles during the cue are counted as they reflect attempted ARs.

Data are presented below for acquisition and extinction for each renewal condition (see Figure 2). Acquisition data considering only avoidance responses (ARs) and not escape responses (ERs) were analyzed using a 3 (Renewal Condition: ABA, AAB or ABC) x 2 (Stimulus: tone vs. noise) x 5 (Day) mixed factorial analysis of variance (ANOVA). There was no significant main effect of Renewal Condition, *F*_(2, 45)_ = 0.410, *p* > 0.05. However, there was a significant main effect of stimulus-type, *F*_(1, 45)_ = 51.754, *p* < 0.001. Simple contrasts revealed that, as observed in prior work (Campese et al., 2017; Fadok et al., 2017), rats in general, shuttled more in response to the white noise (*M* = 21.033) than they did to tone (*M* = 14.7130). Training day also yielded a significant main effect reflecting acquisition of avoidance over this phase, *F*_(4, 180)_ = 11.372, *p* < 0.001. Review of Bonferroni-adjusted pairwise comparisons indicate that the number of shuttle responses significantly increased from acquisition training day 1 to training day 5. Simple contrasts of acquisition training demonstrated that shuttling responses increased most between day 2 (*M* = 14.01) and day 5 (*M* = 19.698) of acquisition training, *F*_(1, 180)_ = 46.906, *p* < 0.001. Of the four potential interaction effects, only the Stimulus x Training day interaction was significant, *F*_(4, 180)_ = 6.556, *p* < 0.001. Further inspection of Bonferroni-adjusted pairwise comparisons and estimated marginal means indicated that, on average, rats shuttled more in response to noise than they did tone on each training day.

Extinction data were analyzed using the same approach and a 3 x 2 x 5 mixed factorial ANOVA was conducted to compare Renewal Condition, Stimulus, and Day, respectively. The main difference in these data is that the AR and ER distinction was rendered meaningless by the extinction contingencies in effect and thus, the analysis considered total CS shuttle responding regardless of when in the cue responding occurred. In this analysis, there was no significant main effect of the between-subjects variable of Renewal, *F*_(2, 45)_ = 0.682, *p* > 0.05. In contrast, there was again, a significant main effect of Stimulus, *F*_(1, 45)_ = 65.452, *p* < 0.001. As previously found, an examination of both estimated marginal means and simple contrasts further detailed that rats shuttled more in response to noise (*M* = 11.967) than they did to tone (*M* = 6.571). Finally, there was a significant main effect of Day, *F*_(4, 180)_ = 38.054, *p* < 0.001 (see Figure 2). As was the case with the acquisition analysis, only the two-way interaction between Stimulus and Day was significant, *F*_(4, 180)_ = 57.413, *p* < 0.001. Inspection of estimated marginal means and Bonferroni-adjusted pairwise comparisons illustrated that although shuttle responding did steadily decrease from extinction training Day 1 to Day 5, rats shuttled more in response to noise during extinction training than they did to tone.

**Figure 3:**
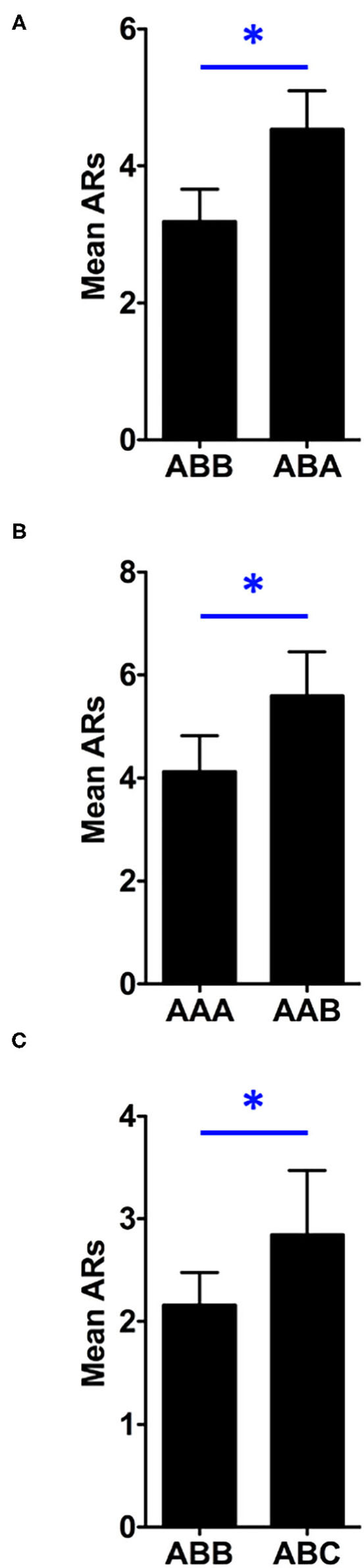
Test data are collapsed across tone and noise and expressed as a function of the stimulus role during each test resulting in the comparisons of ABB vs. ABA **(A)**, AAA vs. AAB **(B)** and ABB vs. ABC **(C)**. Similar to the extinction data, all shuttles during the CSs were counted since no response contingencies were in effect (i.e., the tests were conducted under extinction). Asterisks represent significance at the 0.05 alpha level and error bars, SEM.

To permit analysis of the contextual control over responding following extinction, a preliminary 3 (Renewal Condition) x 2 (Stimulus) x 2 (Test) mixed factorial ANOVA was conducted to compare the renewal behavior outcomes of Test 1 and Test 2. This analysis revealed a significant main effect of between-subjects factor, Renewal Condition, *F*_(2, 45)_ = 5.136, *p* = 0.010. as well as a significant main effect of within-subjects factor of Test, *F*_(1, 45)_ = 23.386, *p* < 0.001. Estimated marginal means indicated that overall responding was greater in Test 2 (*M* = 4.719) than in Test 1 (*M* = 2.760). There was also a main effect of the within-subjects factor, Stimulus, *F*_(1, 45)_ = 9.999, *p* = 0.003. None of the possible interactions were significant in this analysis. Additionally, an analysis was run comparing the effect of context over tests as a function of the renewal assignment (ABA, AAB or ABC) to determine if there were any differences regarding how these different groups expressed renewal over the test phase. This analysis found that there was no difference in the effect of context for the different renewal conditions *F*_(2, 45)_ = 0.431, *p* = 0.65. Therefore, we collapsed across the factors of Test and Stimulus to express the data based on whether the cues were tested in the extinction or non-extinction context for each renewal condition (see Figure 3).

A 3 (Renewal Condition: ABA, AAB or ABC) x 2 (Context: extinction vs. non-extinction) mixed factorial analysis of variance (ANOVA) found a significant main effect of the between subjects factor, Renewal Condition, *F*_(2, 45)_ = 5.136, *p* = 0.010. Estimated marginal means indicated that rats shuttled least when tested for ABC renewal (*M* = 2.50), followed by ABA renewal (*M* = 3.859), and lastly, AAB renewal (*M* = 4.859). To further clarify, overall responding in the AAB condition was highest, and lowest in the ABC condition, but there were not any differences in the strength of these renewal effects themselves. There was also a significant main effect of within-subjects factor, Context, *F*_(1, 45)_ = 9.999, *p* = 0.003. Estimated marginal means illustrated that rats shuttled more when stimuli were tested outside of the extinction context (*M* = 4.323) than they did when tested within the extinction context (*M* = 3.156). There was no significant interaction effect between Renewal Condition and Context, *F*_(2, 45)_ = 0.431, *p* > .05.

## Discussion

The results of this study demonstrate ABA, AAB and ABC renewal of extinguished instrumental avoidance behavior. There have been a limited number of studies that directly assess this, in rodents (Nakajima, 2014) and in humans (Krypotos et al., 2013; Schlund et al., 2020). Moreover, while many studies of even basic Pavlovian renewal fail to adequately control for context-US imbalances, the current study used a well-controlled within-subjects design that equated the associative status of the test contexts by conducting extinction in both locations. Thus, differences in responding (i.e., renewal) can be more easily interpreted as indicating that the cues possessed a different meaning in each context with regards to the avoidance contingencies in effect. With this approach, locations where a specific cue-avoidance contingency was reduced selectively lowered responding to that cue, while preserving avoidance responding to the other cue. The same was true when the roles of these cues were reversed by testing in the alternate context. It should be noted that while studies of purely Pavlovian learning show that the forms of renewal studied here are normally expressed with differing degrees of intensity (i.e., ABA renewal is usually stronger than ABC, which typically exceeds AAB renewal; Rescorla, 2008). However, this was not found in the current study when using an avoidance response as all renewal forms were equally strong.

As previous studies have shown, shuttle behavior appears more strongly motivated in response to white noise rather than tone auditory events. This stimulus difference was also evident in the current study. When this is accounted for by counterbalancing (as was done here), this unconditioned difference becomes less important. However, in procedures that do not evaluate behavior when stimuli play different roles, potential problems of interpretations arise due to these stimulus-specific effects (Fadok et al., 2017). Because tone and noise in the current study were each given similar treatments and the test data were organized based on the role both these stimuli possessed in the different contexts, the data can be understood as reflecting hierarchical control over avoidance responding based on specific associative status each cue held in each context at the time of testing.

While avoidance research is currently experiencing a revival, many unresolved questions as to the nature of this form of learning remain. Given the impressive advancements provided by past work on the core elements of avoidance, specifically aversive Pavlovian conditioning and its extinction, we are in a better position to gather a clear understanding of this complex and historically controversial phenomenon than before (Krypotos et al., 2013; LeDoux et al., 2017; Cain, 2019). While some of this work aims to apply this analysis to treatments of obsessive-compulsive disorder to understand how effective extinction might be at reducing excessive avoidance, another goal is to identify more effective means of attenuating maladaptive fear and anxiety in humans. However, the extent to which avoidance may provide a more viable and effective treatment option for humans suffering from these kinds of disorders is not clear. An established factor in the emergence of avoidance behavior is the reduction of Pavlovian defensive responses such as freezing (Moscarello and LeDoux, 2013; Diehl et al., 2018). Because avoidance training involves a fair amount of CS exposure, it is not surprising to find that the reduction in CRs as avoidance progresses depends on circuits that have also been found to control extinction. Indeed, recent studies have reported that avoidance itself appears limited and is context specific in a similar manner to extinction (Oleksiak et al., 2021). It should be noted that video footage was not recorded during extinction sessions, and the possibility that extinction of avoidance may have caused a recovery of Pavlovian freezing CRs was not explored. The current study only measured shuttle responding because both Pavlovian and instrumental associations were extinguished. A closer analysis of how shuttling and freezing are expressed in a more tightly controlled instrumental contingency degradation study could provide insight as to whether freezing might return in a manner seen in counterconditioning or reversal learning studies involving extinction (Scarlet et al., 2009).

The current study adds to others that extend this analysis to how extinction and avoidance interact under a variety of circumstances to better understand exactly how interdependent they are (Nakajima, 2014; Campese et al., 2017). These studies show that extinction, rather than deepening the effects of avoidance learning, seems to generate identical recovery effects as do basic Pavlovian procedures. If avoidance depends on extinction to remove the competing response, one might expect that Pavlovian extinction might further reduce freezing and allow for stronger expression of the avoidance response. While this did not happen, the current results may also provide evidence against the idea that avoidance transitions into a S[R-O] representation, similar to instances of occasion-setting, or discriminative control. Studies have found that extinction given these kinds of associative representations does not produce a decrement in hierarchical control (Fraser and Holland, 2019; but also see Trask et al., 2017), however, avoidance is very clearly susceptible to extinction and recovery effects (Nakajima, 2014; Campese et al., 2017). Thus, while avoidance has some attractive characteristics insofar as treatment options are concerned, a more controlled approach based on generalization from response learning to stimulus control may provide a better path forward (see Campese, 2021 for a review). The use of transfer testing and separately training Pavlovian and instrumental responding effectively controls for stimulus exposure and potential context-CS associations that are known to influence the different forms of renewal.

## Data Availability Statement

The raw data supporting the conclusions of this article will be made available by the authors, without undue reservation.

## Ethics Statement

The animal study was reviewed and approved by New York University Animal Welfare Committee.

## Author Contributions

VC designed research and wrote manuscript. LB ran studies and helped write manuscript. JL helped write manuscript. All authors contributed to the article and approved the submitted version.

## Funding

NIDA Grant R01 DA044445 and NIMH Grant R01 MH038774 awarded to JL supported this research.

## Conflict of Interest

The authors declare that the research was conducted in the absence of any commercial or financial relationships that could be construed as a potential conflict of interest.

## Publisher's Note

All claims expressed in this article are solely those of the authors and do not necessarily represent those of their affiliated organizations, or those of the publisher, the editors and the reviewers. Any product that may be evaluated in this article, or claim that may be made by its manufacturer, is not guaranteed or endorsed by the publisher.

## References

[B1] Bouton M. E. (2004). Context and behavioral processes in extinction. Learn. Mem. 11, 485–494. 10.1101/lm.78804 15466298

[B2] Bouton M. E. Maren S. McNally G. P. (2021). Behavioral and neurobiological mechanisms of pavlovian and instrumental extinction learning. Physiol. Rev. 101, 611–681. 10.1152/physrev.00016.2020 32970967 PMC8428921

[B3] Cain C. K. (2019). Avoidance problems reconsidered. Curr. Opin. Behav. Sci. 26, 9–17. 10.1016/j.cobeha.2018.09.002 30984805 PMC6456067

[B4] Campese V. D. (2021). The lesser evil: pavlovian-instrumental transfer and aversive motivation. Behav. Brain Res. 412:113431. 10.1016/j.bbr.2021.113431 34175357

[B5] Campese V. D. Delamater A. R. (2013). ABA and ABC renewal of conditioned magazine approach are not impaired by dorsal hippocampus inactivation or lesions. Behav Brain Res. 248, 62–73. 10.1016/j.bbr.2013.03.044 23583520 PMC3763961

[B6] Campese V. D. Kim I. T. Rojas G. LeDoux J. E. (2017). Pavlovian extinction and recovery effects in aversive pavlovian to instrumental transfer. Front. Behav. Neurosci. 11:179. 10.3389/fnbeh.2017.00179 28993726 PMC5622165

[B7] Choi J. S. Cain C. K. LeDoux J. E. (2010). The role of amygdala nuclei in the expression of auditory signaled two-way active avoidance in rats. Learn. Mem. 17, 139–147. 10.1101/lm.1676610 20189958 PMC2832923

[B8] Diehl M. M. Bravo-Rivera C. Rodriguez-Romaguera J. Pagan-Rivera P. A. Burgos-Robles A. Roman-Ortiz C.. (2018). Active avoidance requires inhibitory signaling in the rodent prelimbic prefrontal cortex. Elife 7:e34657. 10.7554/eLife.34657 29851381 PMC5980229

[B9] Fadok J. P. Krabbe S. Markovic M. Courtin J. Xu C. Massi L.. (2017). A competitive inhibitory circuit for selection of active and passive fear responses. Nature 542, 96–100. 10.1038/nature21047 28117439

[B10] Fraser K. M. Holland P. C. (2019). Occasion setting. Behav Neurosci. 133, 145–175. 10.1037/bne0000306 30907616 PMC6447318

[B11] Iordanova M. D. Good M. A. Honey R. C. (2008). Configural learning without reinforcement: integrated memories for correlates of what, where, and when. Q. J. Exp. Psychol. (Hove) 61, 1785–1792. 10.1080/17470210802194324 18609388

[B12] Ji J. Maren S. (2005). Electrolytic lesions of the dorsal hippocampus disrupt renewal of conditional fear after extinction. Learning and Memory. 12, 270–276. 10.1101/lm.91705 15930505 PMC1142455

[B13] Krypotos A. M. Effting M. Arnaudova I. Kindt M. Beckers T. (2013). Individual differences in discriminatory fear learning under conditions of ambiguity: a vulnerability factor for anxiety disorders? Front. Psychol. 4:298. 10.3389/fpsyg.2013.00298 23755030 PMC3664781

[B14] LeDoux J. E. Moscarello J. Sears R. Campese V. (2017). The birth, death and resurrection of avoidance: a reconceptualization of a troubled paradigm. Mol. Psychiatry. 22, 24–36. 10.1038/mp.2016.166 27752080 PMC5173426

[B15] Lissek S. Powers A. S. McClure E. B. Phelps E. A. Woldehawariat G. Grillon C.. (2005). Classical fear conditioning in the anxiety disorders: a meta-analysis. Behav. Res. Ther. 43, 1391–1424. 10.1016/j.brat.2004.10.007 15885654

[B16] Maren S. Holmes A. (2016). Stress and fear extinction. Neuropsychopharmacology 41, 58–79. 10.1038/npp.2015.180 26105142 PMC4677122

[B17] Morgan M. A. LeDoux J. E. (1995). Differential contribution of dorsal and ventral medial prefrontal cortex to the acquisition and extinction of conditioned fear in rats. Behav. Neurosci. 109, 681–688. 10.1037/0735-7044.109.4.681 7576212

[B18] Moscarello J. M. LeDoux J. E. (2013). Active avoidance learning requires prefrontal suppression of amygdala-mediated defensive reactions. J. Neurosci. 33, 3815–3823. 10.1523/JNEUROSCI.2596-12.2013 23447593 PMC3607300

[B19] Nakajima S. (2014). Renewal of signaled shuttle box avoidance in rats. Learn. Motiv. 46, 27–43. 10.1016/j.lmot.2013.12.002

[B20] Oleksiak C. R. Ramanathan K. R. Miles O. W. Perry S. J. Maren S. Moscarello J. M. (2021). Ventral hippocampus mediates the context-dependence of two-way signaled avoidance in male rats. Neurobiol. Learn. Mem. 183:107458. 10.1016/j.nlm.2021.107458 34015439 PMC8319050

[B21] Quirk G. J. Russo G. K. Barron J. L. Lebron K. (2000). The role of ventromedial prefrontal cortex in the recovery of extinguished fear. J. Neurosci. 20, 6225–631. 10.1523/JNEUROSCI.20-16-06225.2000 10934272 PMC6772571

[B22] Rajbhandari A. K. Tribble J. E. Fanselow M. S. (2017). “Neurobiology of fear memory,” in Mechanisms of Memory, Vol. 4 of Learning and Memory: A Comprehensive Reference, 2nd Edn, ed S. J. Sarapp (Oxford: Academic Press), 487–503. 10.1016/B978-0-12-809324-5.21100-0

[B23] Rescorla R. A. (2008). Within-subject renewal in sign tracking. Q. J. Exp. Psychol. (Hove) 61, 1793–1802. 10.1080/17470210701790099 18609366

[B24] Scarlet J. Campese V. D. Delamater A. R. (2009). Sensory-specific associations in flavor preference reversal learning. Learn. Behav. 37, 179–187. 10.3758/LB.37.2.179 19380895

[B25] Schlund M. W. Ludlum M. Magee S. K. Tone E. B. Brewer A. Richman D. M. (2020), Renewal of fear avoidance in humans to escalating threat: Implications for translational research on anxiety disorders. J. Expert Anal. Behav. 113, 153–171. 10.1002/jeab.565. PMC8168406 31803943

[B26] Trask S. Thrailkill E. A. Bouton M. E. (2017). Occasion setting, inhibition, and the contextual control of extinction in Pavlovian and instrumental (operant) learning. Behav. Processes. 137, 64–72. 10.1016/j.beproc.2016.10.003 27720958 PMC5768202

[B27] Zhou W. Crystal J. D. (2009). Evidence for remembering when events occurred in a rodent model of episodic memory. Proc. Natl. Acad. Sci. U. S. A. 106, 9525–9529. 10.1073/pnas.0904360106 19458264 PMC2695044

